# Oral Microbiome in Orthodontic Acrylic Retainer

**DOI:** 10.3390/polym14173583

**Published:** 2022-08-30

**Authors:** Punnisa Kasibut, Jintakorn Kuvatanasuchati, Boonyanit Thaweboon, Irin Sirisoontorn

**Affiliations:** 1 Department of Clinical Dentistry, Walailak University International College of Dentistry (WUICD), 87 Ranong 2 Road, Dusit, Bangkok 10300, Thailand; 2 Department of Oral Health Science, Walailak University International College of Dentistry (WUICD), 87 Ranong 2 Road, Dusit, Bangkok 10300, Thailand

**Keywords:** oral microbiome, orthodontic, acrylic retainer

## Abstract

The oral microbiome can be shifted if the patients wear the acrylic retainers for a lengthy period. It is essential to understand the components of the plaque in order to forestall the development of dental caries and gingivitis. The aim of this study is to report the bacterial communities that adhere to the acrylic retainers by full-length nanopore 16S sequencing. Six healthy participants were allocated into 2 groups (chemical tablet and brushing groups). Plaque samples were collected from the acrylic retainer surfaces before and after cleaning. The bacterial communities were reported using full-length nanopore 16S sequencing. The results showed that 7 distinct phyla were identified by sequencing. The most prevalent of these was the Firmicutes. We found a total of 72 genera. The most common microorganism across all samples was *Streptococcus*, followed by *Neisseria*, *Rothia*, and *Gemella*. The beta diversity showed a significant difference between before and after cleaning (*p* < 0.05). This study revealed the novel finding that a combination of chemical and mechanical cleaning methods was the most effective method of eliminating retainer biofilms. Moreover, retainer cleaning tablets did not alter the homeostatic balance of the bacterial communities adhering to the acrylic retainers.

## 1. Introduction

Generally, wearing the retainer is required for the retention phase to preserve the position of the teeth after orthodontic treatment has been completed [1]. Retainers, either fixed or removable appliances, will be used for retention. Removable wrap-around acrylic retainers are the most common type of the acrylic retainers worn following orthodontic treatment [2]. Acrylic retainers are notorious for bacterial colonization due to their porous acrylic baseplates [3]. The recommended daily wear time for the retainers is 8–12 h [4]. Dental caries and gingival irritation can develop after lengthy retainer use because of a deterioration in oral hygiene and a shift in the microbial flora [5]. In previous research, *Streptococcus mutans* and *Lactobacillus* developed in individuals who wore removable orthodontic devices for 6 months [6]. Moshiri et al. reported gingival irritation and tooth decay in a teenage male who wore a removable appliance without washing it for 4 months [7].

According to Drogramaci et al., bacterial growth promotion caused carious lesions and denture-like stomatitis [8]. Moreover, a study by Shi et al. found a positive correlation between *A. parvulum*, *Lachnospiraceae* sp., *Veillonella atypica* and *C. albican* on the dentures of stomatitis patients [9]. Therefore, the acrylic retainers should be cleaned regularly to avoid developing carious lesions and gingivitis. Cleaning acrylic retainers can be classified into two methods: mechanical and chemical methods. An example of a mechanical method is brushing, which can be performed with or without toothpaste. Hypochlorites, peroxides and acids are just a few examples of how chemical processes can be categorized according to their composition and mechanisms of action. However, only a few chemicals are safe to clean the acrylic retainers [10]. Recently, there is a commercial chemical tablet for cleaning retainers, but there is only a little evidence to support its efficiency and its effect on species diversity of bacteria on the surfaces of the acrylic retainers.

Patients’ oral health can be jeopardized by the development of biofilms adhering to the surfaces of the acrylic retainers [11]. Understanding the etiology of a disease requires familiarity with the microbiota that inhabits biofilms. To better understand the microbiome’s potential role in carious lesions and other oral disorders, Oxford Nanopore Technologies (ONT) (Oxford, UK) has been applied to the field of oral microbiology [12]. To further understand the diversity of the microbial biofilms adhering to the acrylic retainers, we investigated their full-length 16S ribosomal ribonucleic acid (rRNA) gene sequences (V1–V9). Full-length sequencing of the bacterial 16S rRNA gene was used in this research. The effectiveness of the chemical tablets was compared to that of mechanical procedures, and the species diversity difference was determined by alpha and beta analysis. To the best of our knowledge, this preliminary study revealed the first report of the bacterial communities adhering to the acrylic retainers at the species level.

## 2. Materials and Methods

### 2.1. Study Design and Participants

The study protocol (WUEC 1-507-63) was granted an exemption by the Walailak University Committee for Ethics in Human Research before selecting participants therefore this exemption had been reviewed and approved. Six participants (2 males and 4 females) were selected at random from the patient rosters of a private dental clinic in Thailand, with ages ranging from 30 to 59, all of whom had been wearing the acrylic retainers for at least 6 months. Six patients’ retainers were used in this study.

Inclusion Criteria [13]:

- Wear the acrylic retainer for 24 h.
- Wear the acrylic retainer for more than 6 months, except when eating.
- Nonsmoker.
- Without any medication that reduces saliva flow.
- All acrylic retainers are fabricated in the same laboratory.

Exclusion Criteria:

- Use of steroid-based or antibacterial mouthwash or broad-spectrum antibiotics for 2 weeks before the study.

**Figure 1:**
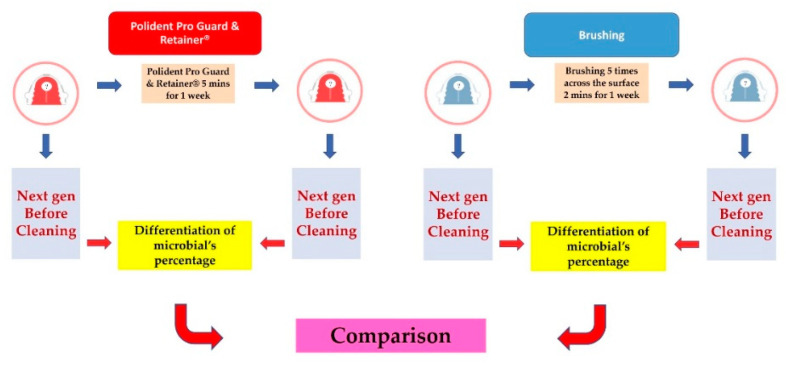
Study design.

Patients were randomly divided into 2 equal groups. Each group represented different cleaning methods, as shown in Figure 1. Sample 1, 2 and 3 represented the chemical tablet group. Sample 4, 5 and 6 represented the brushing group. Plaque collection was performed before and after using the chemical tablets also before and after brushing. Chemical tablet group was called 1Before (1B), 1After (1A), 2B, 2A, 3B and 3A. Brushing group was called 4Before (4B), 4After (4A), 5B, 5A, 6B and 6A. 

### 2.2. Cleaning the Acrylic Retainers

For the chemical tablet group (1A, 2A and 3A), the acrylic retainers were cleaned by Polident Pro Guard & Retainer^®^ in accordance with the manufacturer’s instructions. Briefly, the acrylic retainers were immersed in Pro Guard & Retainer^®^ solution for 5 min and removed immediately. These procedures were repeated for 7 days before plaque collection. For the brushing group (4A, 5A and 6A), the acrylic retainers were brushed 5 times across the surfaces using a toothbrush for 2 min every day for 1 week [14].

### 2.3. Plaque Collection

Plaque samples were collected from the acrylic retainer surfaces by using sterile toothpicks in a circular motion [9]. Individual plaque samples were placed into separate sterile 1.5 mL tubes with saline solution and immediately kept at −20 °C until DNA extraction [15].

### 2.4. DNA Extraction

Using the PureLink™ Genomic DNAMini Kit (Invitrogen™, Waltham, MA, USA) for extracting deoxyribonucleic acid (DNA). The procedure was performed following the manufacturer’s standard protocol. After extraction, DNA was measured using a Nanodrop lite spectrophotometer (Thermo Scientific, Waltham, MA, USA) and stored at −30 °C.

### 2.5. DNA Preparation and 16S Microbiome Sequencing

The total DNA was extracted using ZymoBIOMICS™ DNA Miniprep Kit following the manufacturer’s instructions. The full-length bacterial 16s rDNA (~1500 bp) was amplified by PCR using the tailed primers containing Nanopore PCR adaptor: 5′-TTTCTGTTGGTGCTGATATTGCAGRGTTYGATYMTGGCTCAG-3′ and 5′-ACTTGCCTG TCGCTCTATCTTCCGGYTACCTTGTTACGACTT-3′ modified from the previous study [16]. The 20 µL PCR reaction contained 10 ng of DNA template, 1X Phusion™ Plus buffer (Thermo scientific, Waltham, MA, USA), 0.25 µM of each primer, 0.2 mM of dNTPs and 0.2 µL of Phusion™ Plus DNA Polymerase (Thermo scientific, Waltham, MA, USA). The PCR amplification was performed using following thermal conditions: 98 °C for 30 s, 25 cycles of 98 °C for 10 s, 60 °C for 25 s, 72 °C for 45 s and 72 °C for 5 min. After that, the amplicons were barcoded by 5-cycles PCR using the barcode primers based on PCR Barcoding Expansion 1–96 (EXP-PBC096) kit (Oxford Nanopore Technologies, Oxford, UK). The amplified products were examined using 1% agarose gel electrophoresis and purified using QIAquick^®^ PCR Purification Kit (QIAGEN, Hilden, Germany). The DNA products were pooled equimolarly and subsequently purified using 0.5× Agencourt AMPure XP beads (Beckman Coulter, Brea, CA, USA). Then, the purified library was end-repaired and adaptor-ligated using Ligation Sequencing Kit (SQK-LSK112) (Oxford Nanopore Technologies, Oxford, UK). Finally, the library was loaded onto the R10.4 flow cell and sequenced on MinION Mk1C sequencer.

### 2.6. Data and Statistical Analysis

The FAST5 data were basecalled by guppy basecaller v6.0.1 (Oxford Nanopore Technologies, UK) with the super-accuracy model to generate the FASTQ files. The quality of reads was examined by MinIONQC [17]. Then, FASTQ sequences were demultiplexed and adaptor-trimmed using Porechop v0.2.4.

The filtered reads were then clustered, polished and taxonomically classified by NanoCLUST [18] based on the reference sequences from Ribosomal Database Project (RDP) database v11.5. The bacterial abundance and diversity analysis were analyzed and illustrated by MicrobiomeAnalyst [19].

## 3. Results

### 3.1. Demographic and Clinical Information

As shown in Table 1, the retainer plaques were taken from 6 healthy participants (no carious lesion, no periodontal lesion and not overweight).

### 3.2. Phylum, Genus and Species Composition of Biofilms adhering to the Acrylic Retainers

Biofilm samples from the acrylic retainers were split into 2 groups for DNA extraction: those treated with chemical tablets (3 samples) twice before and after, and those treated with mechanical methods (3 samples) twice before and after. The full-length 16S gene allowed for better taxonomic resolution. The 1500-bp 16S rRNA gene was divided into 9 parts, all of which had the same stable 16S sequence. Sequencing was performed on a total of 12 samples, which produced 105,316 reads with an average of 8776 and a median of 8444.5 (range: 6605–12,184).

**Figure 2:**
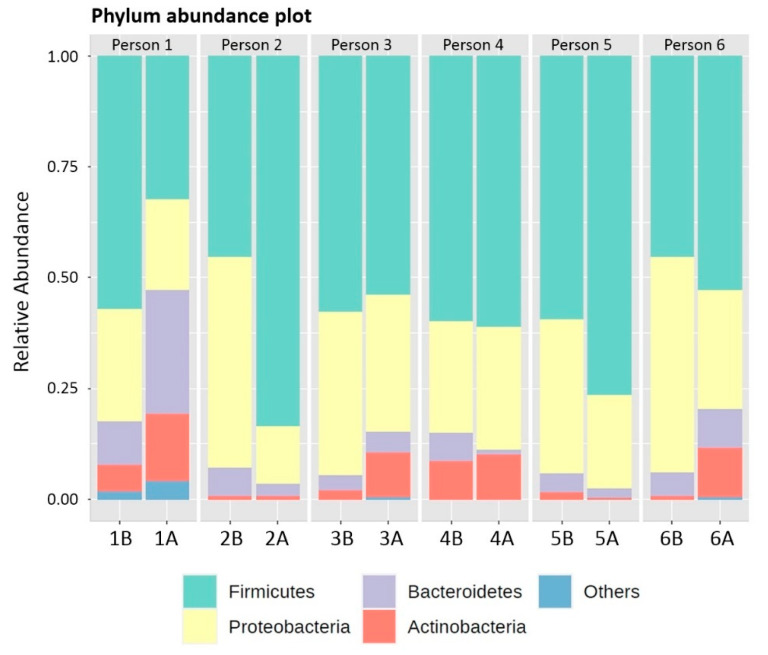
Relative abundance (%) of detected phyla.

There were only 4 different phyla that could be identified in all 4 groups of retainer samples including Actinobacteria, Bacteroidetes, Firmicutes and Proteobacteria (Table 2). Interestingly, Proteobacteria was the only phylum that decreased after the cleaning. Spirochaetes and Synergistetes were found only in 1A. Fusobacteria was found only in 1B, 1A, 3A and 6A. Relative abundances (%) of detected phyla showed that Firmicutes was the most common phylum and could be found in all 12 samples (Figure 2).

**Figure 3:**
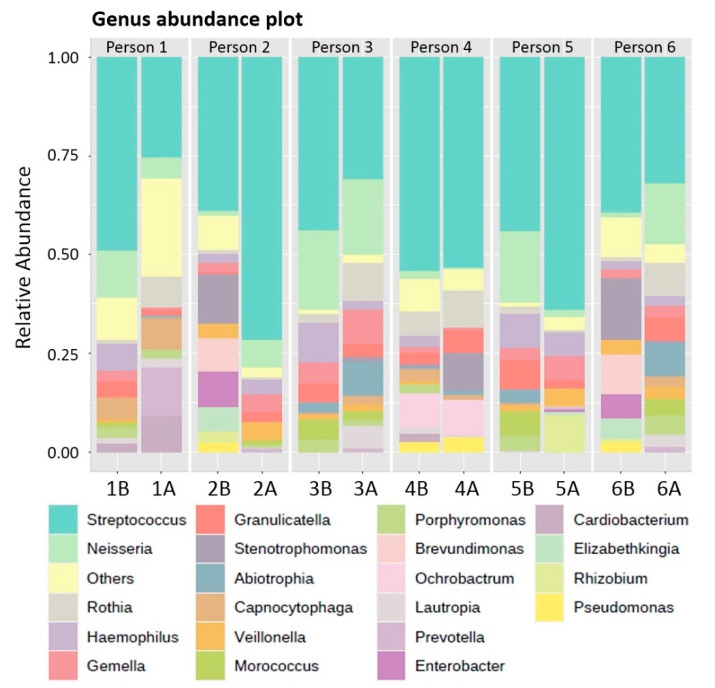
Relative abundance (%) of detected genera.

**Figure 4:**
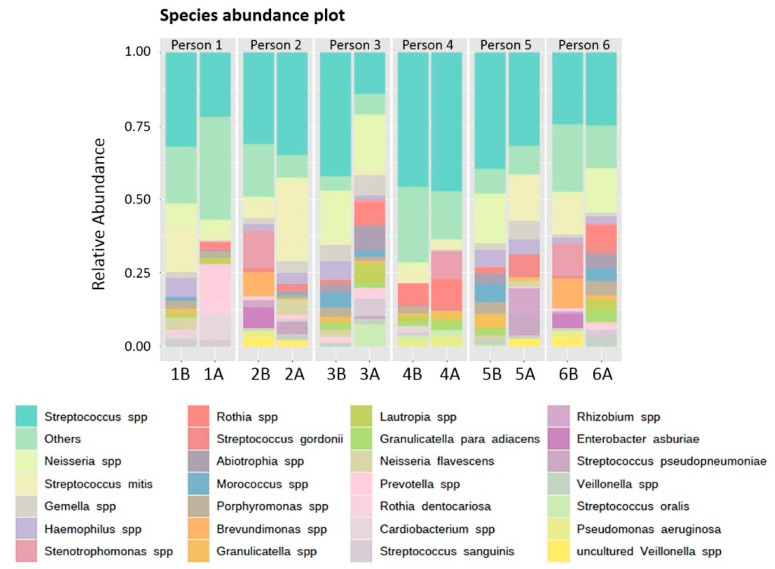
Relative abundance (%) of detected species.

Moreover, we discovered a total of 72 genera: 47 genera in the chemical tablet group (before), 41 genera in the chemical tablet group (after), 34 genera in the brushing group (before) and 39 genera in the brushing group (after). The most common microorganism across all samples was *Streptococcus*, followed by *Neisseria, Rothia* and *Gemella* (Figure 3). A total of 139 different species were identified. *Streptococcus mitis* was the most common species across all taxonomic categories, followed by *S. gordonii*, *N. flavescens*, *S. sanguinis* and *R. dentocariosa* (Figure 4).

### 3.3. Diversity of Bacterial Composition on Biofilms of the Acrylic Retainers

**Figure 5:**
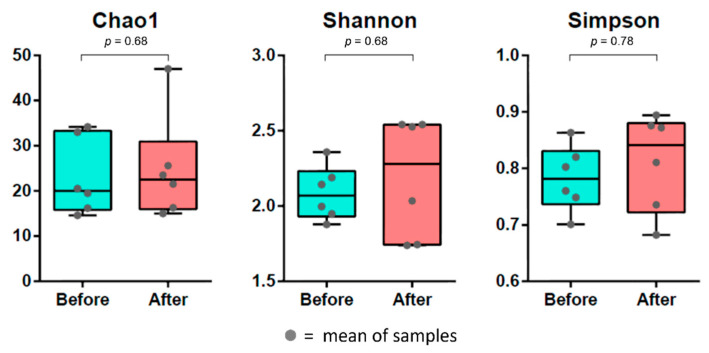
Alpha diversity compared between before and after cleaning the acrylic retainers.

**Figure 6:**
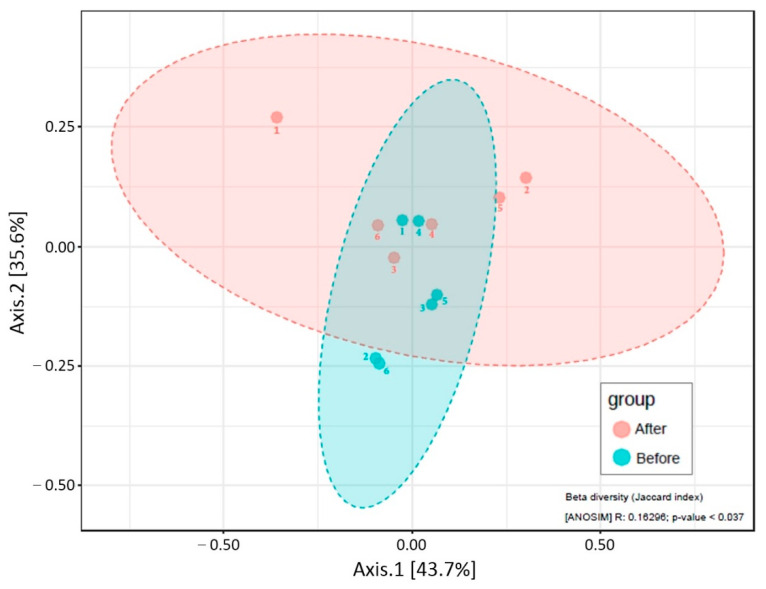
Beta diversity compared between before and after cleaning the acrylic retainers.

**Figure 7:**
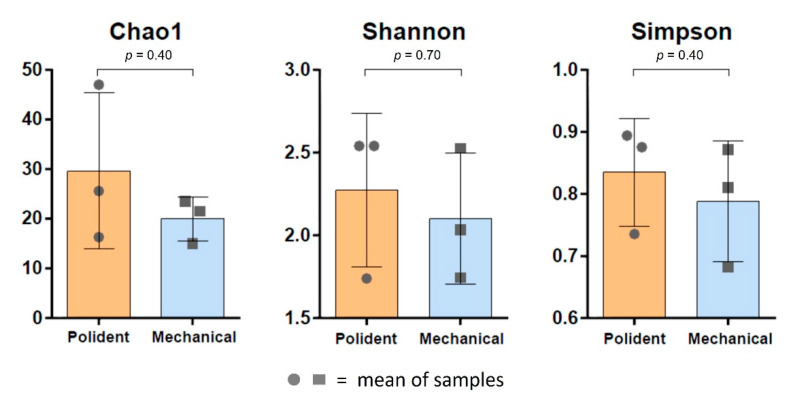
Alpha diversity compared between the chemical tablet and the brushing groups.

The diversity index was used to find variations between groups of bacterial species. The results of Chao1, Shannon and Simpson index measurements including statistical analysis were used to quantify species diversity between groups. The alpha diversity indexes showed that there was no significant difference between before and after cleaning groups, as shown in Figure 5. However, beta diversity showed a significant difference between before and after cleaning groups (*p* < 0.05), as shown in Figure 6. In addition, there was no significant difference between the chemical tablet and the brushing groups, as shown in Figure 7.

## 4. Discussion

A different method for cleaning the acrylic retainers has been created for reducing a multi-species retainer biofilm. There is still no consensus on the proper cleaning regimen [20]. Inadequate retainer cleaning can also encourage biofilm formation, which can exacerbate oral mucosal inflammation. Our beta diversity analysis demonstrated that both groups showed significantly different in species diversity when chemical and mechanical cleaning procedures were applied, compared to the initial state. This research revealed the novel finding that both chemical and mechanical cleaning methods could affect species diversity of bacterial communities adhering to the acrylic retainers. Moreover, our study was the most recent clinical study to compare biofilm adhering to the acrylic retainers that used full-length 16S rRNA gene sequencing on platforms developed by ONT for the V1–V9 regions. Short-read sequencing can significantly reduce the taxonomic precision of 16S rRNA sequencing, which can only typically resolve down to the genus level. When a gene is sequenced in its entirety, all 1500 base pairs (bp), more taxonomic detail can be obtained than when using short-read sequencing techniques. For these reasons, we used full-length to obtain the result at the species level. This notion was supported by previous study showing that full-length 16S rRNA reads gave better taxonomic resolution than reading only hypervariable regions [21].

The primary purpose of this study is to document the types and relative abundances of bacteria that adhere to the acrylic retainers. Across all samples, the phyla Firmicutes, Bacteroidetes, Proteobacteria and Actinobacteria were well represented. The oral flora from all these phyla were related to earlier studies, indicating the bacterial diversity of the microflora in 9 distinct places among 5 clinically healthy participants. Six phyla were identified in this study as follows: the Firmicutes (*Streptococcus*, *Gemella*, *Eubacterium*, *Selenomonas*, *Veillonella* and related ones), the Actinobacteria (*Actinomyces*, *Atopobium*, *Rothia*, etc.), the Proteobacteria (*Neisseria*, *Eikenella*, *Campylobacter* and related ones), the Bacteroidetes (*Capnocytophaga*, *Porphyromonas*, *Prevotella*, etc.), the Fusobacteria (*Leptotrichia* and *Fusobacterium*) and the phylum of TM7. Firmicutes are the most phyla in the retainer’s plaque. When many Firmicutes are found in the mouth, it means that polysaccharide hydrolysis has begun to occur [22]. In addition, Firmicutes also play a significant role in the relationship between gut bacteria and human health. Firmicutes are important to human health because they help the body absorb fats and break down lipids [23].

Recently, the Firmicutes/Bacteroidetes (F/B) ratio has been widely considered crucial in maintaining appropriate intestinal homeostasis. Dysbiosis is described as either an increase or a drop in the F/B ratio; this situation is often seen with obesity or inflammatory bowel illness (IBD) [24]. The F/B ratio and body mass index (BMI) are substantially correlated. Those persons with a F/B ratio ≥ 1 were 23% more likely to be overweight than those with a F/B ratio < 1 therefore Firmicutes are representative of obesity in the gut [25]. However, our study found that F/B ratio was greater than 1 but did not correspond to being overweight as defined by BMI. The microbial diversity in the oral cavity may differ from the gut which could affect the F/B ratio. According to findings from earlier study, only Firmicutes were associated with active dental caries, whereas Proteobacteria and Fusobacteria did not have any relation. To avoid the progression of dental caries, several phyla play preventative functions such as blocking pathogen colonization and counteracting its metabolism [12]. Based on our findings, a reduction in Proteobacteria was seen in both groups after cleaning, however, Polident Pro Guard & Retainer^®^ had no effect on the number of Fusobacteria. Moreover, we found that *Streptococcus* was the most common genus adhering to the acrylic retainers. Contrary to earlier research, Actinomyces have been determined to be the most common denture-associated bacteria [9]. 

The plaque that adhere to the acrylic retainers is multi-species. Bacteria and *Candida albicans* are only two examples of opportunistic infections that adhere to the acrylic retainers and lead to either a localized or systemic infection [26]. Prosthodontic applications of polymethyl methacrylate (PMMA) are widely used for dentures [27,28] and the acrylic retainers despite the fact that the rough surfaces and porosity make it easy for *C. albicans* and other microorganisms to build biofilms [3]. Furthermore, it appears that interactions between kingdoms play a significant role in protecting certain bacterial and fungal species from being killed by antibiotics and antifungals. According to a number of studies, there was a favorable correlation between *Candida albicans* and a few different types of bacteria. Their results suggested a positive correlation between *Candida* and *Veillonella atypica*, a *Lachnospiraceae* sp. strain and *A. parvulum*, but *Leptotrichia* sp. was not present in samples containing *Candida* [9]. In correlation analysis, the strong association between *Candida* species and the most common bacterial genera (*Lactobacillus*, *Scardovia* and *Fusobacterium*) was found [29]. Our research showed that using Polident Pro Guard & Retainer^®^ and brushing did not reduce the amount of *Veillonella* spp. in some samples by a large amount.

The oral microbiota changes as a result of orthodontic appliances, with an increase in *S. mutans* and *Lactobacillus* species including a higher proportion of potentially harmful gram-negative bacteria [5]. Focusing on pathogenic bacteria, we identified *Actinomyces viscosus*, *Gordonia bronchialis*, *Propionibacterium propionicum*, *Porphylomonas* spp., *Prevotella loescheii*, *Prevotella nigrescens*, *Capnocytophaga gingivalis*, *Campylobacter gracilis*, *Capnocytophaga granulosa*, *Streptococcus* spp., *Selenomonas noxia*, *Veillonella parvula*, *Fusobacterium nucleatum*, *Fusobacterium periodonticum* and *Pseudomonas aeruginosa*. This finding related to the previous study reporting that the periodontitis-causing pathogens were as follows: *Actinomyces viscosus*, *Porphylomonas* spp., *Prevotella loescheii*, *Prevotella nigrescens*, *Capnocytophaga gingivalis*, *Campylobacter gracilis*, *Capnocytophaga granulosa*, *Selenomonas noxia*, *Veillonella parvula* and *Fusobacterium* [29]. *Streptococcus* spp. and *Veillonella* spp. are identified as causative agents of dental caries [3]. The percentage of *Streptococcus* spp. in samples taken before and after treatment with Polident Pro Guard & Retainer^®^ was found to be 1Before (1B) = 27.46% and 1After (1A) = 15.23% also 3Before (3B) = 41.94% and 3After (3A) = 13.45%, respectively. Consistent with prior findings, this study demonstrated that daily Polident^®^ treatment decreased the number of *Streptococcus* spp. in the biofilm adhering to removable partial denture. Polident Pro Guard & Retainer^®^ has antimicrobial effects on acrylic due to the peroxide in effervescent cleansers, which kills microorganisms. Active oxygen and hydrogen peroxide, which are both antibacterial agents, are included in the mixture along with enzymes degrading the proteins that make up the biofilm [30].

Dental caries and periodontitis are the two most frequent human dental diseases caused by oral flora dysbiosis. Even though commensal bacteria have been shown to defend against infections and boost oral health [31]. Much normal flora involved healthy plaque biofilm adhering to the acrylic retainers. For instance, *S. sanguinis*, which generates H_2_O_2_, can prevent the development of *S. mutans* [32]. *S. sanguinis* was not affected by Polident Pro Guard & Retainer^®^ (1B = 1.44% and 1A = 1.40%). Having *C. durum* around can shield *S. sanguinis* from the host immune response [33]. One sample was focused on *C. durum* showed that Polident Pro Guard & Retainer^®^ was not induced changes to this microflora. Furthermore, *S. gordonii* can inhibit the transcriptional regulator of *Porphyromonas gingivalis*, which is implicated in the inflammatory response of oral epithelial cells [34]. From our study found that there was no effect of Polident Pro Guard & Retainer^®^ on *S. gordonii*. 

## 5. Limitations

The microbial diversity in the oral cavity varies from person to person which could affect the species diversity analysis. Since each species of bacteria has a unique vulnerability to the specific method of cleaning [13], it could possibly limit the species analysis results when compared between different participants. To overcome this limitation, future work should increase the participant population as well as classify participants who share similar bacteria diversity into the analysis.

## 6. Conclusions

In this study, the most effective method of cleaning retainer biofilms was combination therapy that used both Polident Pro Guard & Retainer^®^ and brushing. Moreover, Polident Pro Guard & Retainer^®^ was safe to use because it did not alter the homeostatic balance of the bacterial communities adhering to the acrylic retainers.

## Figures and Tables

**Table 1:** Demographic and Clinical Information.

ID	Age	Gender	Probing Depth	Periodontal Index (PI)	Firmicutes/ Bacteroidetes (F/B)	Body Mass Index (BMI)
1	30	F	≤3	0.83	5.51	19.10
2	33	M	≤3	0.5	7.11	18.73
3	59	F	≤3	0.67	16.57	22.66
4	40	F	≤3	0.75	9.31	19.53
5	33	M	≤3	0.5	11.89	18.75
6	58	F	≤3	0.67	8.56	22.27

**Table 2:** The mean and median of phyla in each group.

Phyla	Chemical Tablet Group (Before)		Chemical Tablet Group (After)		Brushing Group (Before)		Brushing Group (After)	
	Mean	Median	Mean	Median	Mean	Median	Mean	Median
Actinobacteria	2.73%	2.11%	7.51%	9.90%	3.53%	1.63%	7.11%	9.23%
Bacteroidetes	6.18%	6.14%	13.62%	4.53%	5.33%	4.99%	4.88%	3.66%
Firmicutes	50.17%	49.25%	57.11%	56.45%	52.62%	54.38%	60.97%	55.96%
Proteobacteria	40.42%	36.55%	24.84%	16.61%	38.50%	34.77%	26.62%	26.18%

## Data Availability

Not applicable.
